# Appropriate and Constant Potassium Supply Promotes the Growth of M9T337 Apple Rootstocks by Regulating Endogenous Hormones and Carbon and Nitrogen Metabolism

**DOI:** 10.3389/fpls.2022.827478

**Published:** 2022-03-15

**Authors:** Xinxiang Xu, Fen Wang, Yue Xing, Jingquan Liu, Mengxue Lv, Hao Meng, Xin Du, Zhanling Zhu, Shunfeng Ge, Yuanmao Jiang

**Affiliations:** State Key Laboratory of Crop Biology, College of Horticulture Science and Engineering, Shandong Agricultural University, Tai’an, China

**Keywords:** apple rootstock, hormones, K levels, N metabolism, carbon metabolism

## Abstract

Potassium (K) is an indispensable nutrient element in the development of fruit trees in terms of yield and quality. It is unclear how a stable or unstable supply of K affects plant growth. We studied the root morphology and physiological and molecular changes in the carbon and nitrogen metabolism of M9T337 apple rootstock under different K levels and supply methods using hydroponics. Five K supply treatments were implemented: continuous low K (K_L_), initial low and then high K (K_LH_), appropriate and constant K (K_AC_), initial high and then low K (K_HL_), and continuous high K (K_H_). The results showed that the biomass, root activity, photosynthesis, and carbon and nitrogen metabolism of the M9T337 rootstocks were inhibited under K_L_, K_H_, K_LH_ and K_HL_ conditions. The K_AC_ treatment promoted root growth by optimizing endogenous hormone content, enhancing carbon and nitrogen metabolism enzyme activities, improving photosynthesis, optimizing the distribution of carbon and nitrogen, and upregulating the transcription levels of nitrogen assimilation-related genes (nitrate reductase, glutamine synthetase, glutamate synthase, *MdNRT1.1, MdNRT1.2, MdNRT1.5, MdNRT2.4*). These results suggest that an appropriate and constant K supply ensures the efficient assimilation and utilization of nitrogen and carbon.

## Introduction

Potassium (K) is an essential nutrient element for higher plant cells. K plays an important role in maintaining the cell membrane potential gradient, promoting intracellular enzyme activity, regulating photosynthesis, and altering carbon (C) and nitrogen (N) metabolism (Marschner, 2012; Oosterhuis et al., 2014). K addition can also increase the content of phenolic compounds, reduce the occurrence of diseases, and improve the yield and quality of crops (Amtmann et al., 2008; Mirande-Ney et al., 2019). Soil K is the primary source of K absorbed by plants, but most of this is in mineral form. The available K which is directly absorbed and utilized by plants only accounts for only 0.1–2% of the total K content in the soil (Chérel et al., 2014). According to the previous investigations of fertilization in different apple-producing areas in China, the orchard managers generally apply N but ignore phosphorus (P) and K applications (Zhu et al., 2018). The K balance in the soil has gradually been destroyed, and K deficiency in orchard soil has become a common production problem. In recent years, with the rapid development of the apple industry and the resulting economic benefits, orchard managers have realized the importance of K input. Because of the lack of an in-depth understanding of proper fertilization in orchards, excessive and insufficient K fertilizer applications occur (Zheng et al., 2017). Under long-term low or high K conditions, especially under a suboptimal K supply conditions, the soil K supply becomes unstable. The effects on the growth and metabolism of fruit trees remain unclear.

N is a core nutrient for all living organisms including fruit trees (Chen et al., 2018). Because the fruit yield per unit of applied N is higher than that of P and K in most cases, farmers often choose to apply more N fertilizer in pursuit of a higher yield (Zhu et al., 2020). This not only wastes N fertilizer, but also causes a series of environmental problems such as soil acidification and water eutrophication because plants use less than 50% of applied N to the soil (Liu X. J. et al., 2013; Coskun et al., 2017a). Therefore, improving nitrogen use efficiency (NUE) has become a vital issue in fruit production. As counter ions of NO_3_^–^, the absorption of K^+^ and NO_3_^–^ are usually positively correlated and mutually reinforcing (Raddatz et al., 2020). K has high mobility in plants, and K cycling between roots and shoots also plays an important role in the transport of NO_3_^–^ and amino acids in the xylem and phloem (Coskun et al., 2017b). It is well known that the uptake and transport of NO_3_^–^is largely determined by nitrate transporters (NRT) and the NO_3_^–^ can be further transported and utilized by the catalysis of a series of N-metabolizing enzymes (Teng et al., 2017; Morales de Los Rios et al., 2021). K promoted the activities of nitrate reductase (NR), glutamine synthetase (GS), glutamate synthase (GOGAT) and other nitrogen metabolism enzymes, which were verified in cotton (Hu et al., 2016) and cucumber (Ruiz and Romero, 2002). K can provide energy and facilitate N assimilation by increasing photosynthetic C metabolism, promoting protein synthesis and improving NUE (Hu et al., 2017). In addition, K also affects the transport and distribution of N in plants. Generally, the co-translocation of NO_3_^–^ and K^+^ to shoots increases with sufficient K^+^ supply. In recent years, studies have suggested that processes regulating K and N nutrition in plants are closely related at the molecular level (Coskun et al., 2017b). Studies in *Arabidopsis* have shown that K deficiency not only induces the expression of K channel/transporter genes but also downregulates the expression of nitrate transporters NRT1.1, NRT2.1, and NRT1.5 (Armengaud et al., 2009).

Ingestad (1982) proposed the concept of plant steady-state nutrition. An appropriate and constant nutrient supply can resolve problems caused by a high concentration of elements in solution and continuous nutrient depletion, thereby maintaining the maximal growth rates of plants. Imo and Timmer (1992) found that the growth and nutrient status of seedlings can reach a stable state under steady-state nutrient fertilization, which is conducive to growth. Peng et al. (2018) also found that an unstable N supply could influence NUE and inhibit the growth of apple rootstocks. In recent years, excessive N and immoderate K applications have caused low NUE in apple production. In fruit production, there are significant differences in K fertilizer applications among farmers, resulting in evident instability. Although numerous recent studies have examined the relationship between N and K, most have focused on low K stress, and few have investigated the effects of stable and unstable K supply on apple growth and N uptake. Therefore, we analyzed the changes in the physiological index and the absorption, assimilation, and distribution of C and N in apple rootstocks. Our objective was to explore the physiological mechanism of steady or unsteady K supply on apple rootstock growth and N and C metabolism to provide a theoretical basis for the rational application of N and K.

## Materials and Methods

### Experimental Materials and Design

The experiment was conducted in 2020. M9T337 rootstocks, an apple dwarf rootstock, were used in the experiments. The experiment was conducted in a growth chamber with 65% relative humidity under natural light at 28/18°C during the day and at 10/5°C at night. M9T337 rootstocks (about 12 cm in height) were planted in plastic basins (35 cm × 28 cm × 15 cm) containing 6 liters of 1/2 Hoagland’s nutrient solution [2.5 mM Ca(NO_3_)_2,_ 2.5 mM KNO_3_, 1 mM MgSO_4_, 0.5 mM KH_2_PO_4_, 0.1 mM Fe-EDTA, 20 μM H_3_BO_3_, 4.5 μM MnCl_2_, 0.4 μM ZnSO_4_ and 0.2 μM CuSO_4_]. The pH of the nutrient solution was adjusted to 6.0 ± 0.1 with H_3_PO_4_ or NaOH. The solution was replaced every 3 days. When the rootstocks were about 15 cm high, rootstocks with similar growth were selected for the experiment.

Our previous experiment found that the growth of M9T337 rootstocks was the best under a concentration of 6 mM K^+^ (Xu et al., 2020). Therefore, the appropriate K level for this experiment was set at 6 mM. Three K levels were set in the experiment, which were 0.1, 6 and 12 mM respectively. The concentrations of other elements were the same across treatment groups: 5 mM Ca(NO_3_)_2_, 1 mM NaH_2_PO_4_, 2 mM MgSO_4_, 0.1 mM EDTA-Fe, 9 μM MnCl_2_⋅4H_2_O, 37 μM H_3_BO_4_, 0.76 μM ZnSO_4_⋅7H_2_O and 0.3 μM CuSO_4_⋅5H_2_O. Five K supply modes were set up: (1) Keep the K^+^ concentration of nutrient solution at 0.1 mM (continuous low K, K_L_); (2) The concentration of K^+^ in nutrient solution was 0.1 mM in the first 15 days and 12 mM in the last 15 days (low and then high, K_LH_); (3) Keep the K^+^ concentration of nutrient solution at 6 mM (appropriate and constant K supply, K_AC_); (4) The concentration of K^+^ in nutrient solution was 12 mM in the first 15 days and 0.1 mM in the last 15 days (high and then low, K_HL_); (5) Keep the K^+^ concentration of nutrient solution at 12 mM (continuous high K, K_H_). The pH of all nutrient solutions was adjusted to 6.0 ± 0.1 with H_3_PO_4_ or NaOH.

### ^13^C and ^15^N Labeling Method and Isotope Analysis

The rootstocks were labeled with ^13^C after 15, 20, and 25 days of treatment. The rootstocks (10 rootstocks for each treatment) were placed together with the markers (Ba^13^CO_3_, ^13^C independence is 98%, 0.2 g) and fans into a sealed marking room. Labeling work started at 9:00 AM and finished at 1:00 PM. Every 0.5 h, 5 mL of hydrochloric acid (1 mM) was injected into the beaker with a syringe to maintain the concentration of CO_2_. We added an appropriate amount of ice to the bottom of the labeling chamber to control the temperature. Three other plants were selected as the blank control (^13^C natural abundance). Seventy-two hours after the labeling, the samples were destructively sampled for ^13^C determination.

Ten rootstocks (one pot) were selected for ^15^N labeling in each treatment. Replaced 5 mM Ca(NO_3_)_2_ with 4 mM Ca(NO_3_)_2_ and 1 mM Ca(^15^NO_3_)_2_ (with abundance of 10.14%). Other nutrient contents and management are the same as those described above. After 30 days of treatment, the rootstocks were divided into leaves, stems and roots. They were put in paper envelopes and dried at 80°C for 3 days. Then they were ground and filtered with a 0.25 mm mesh screen. The abundance of ^15^N and ^13^C were measured with a MAT-251-Stable Isotope Ratio Mass Spectrometer at the Chinese Academy of Agricultural Sciences (Beijing). Three rootstocks were mixed together for each treatment as a repeat, and each treatment was repeated three times. The ^15^N and ^13^C formula is calculated according to Xu et al. (2020).

Calculation of ^15^N


(1) $$\text{Ndff}\left(\%\right)=\frac{\begin{array}{c} \mathrm{abundance}{\mathrm{of}}^{15}N\mathrm{in}\mathrm{plant}-\\ \mathrm{natural}\mathrm{abundance}{\mathrm{of}}^{15}N \end{array}}{\begin{array}{c} \mathrm{abundance}{\mathrm{of}}^{15}N\mathrm{in}\mathrm{fertilizer}-\\ \mathrm{natural}\mathrm{abundance}{\mathrm{of}}^{15}N \end{array}}\times 100\%$$

(2) N15absorbedbyeachorganfromfertilizer(mg)=Organtotalnitrogen(mg) ×Ndff(%)

(3) N15partitioningrate(%)=N15absorbedbyeachorganfromfertilizer(mg)totalN15absorbedbyplantfromfertilizer(mg)×100%

Calculation of ^13^C


(4) AbundanceofC13:Fi(%)=(δ13C+1000)×RPBD(δ13C+1000)×RPBD+1000×100%

R_PBD_ (standard ratio of carbon isotope) = 0.0112372

Carbon content of each organ: C_i_ = amount of dry matter (g) × total carbon content (%)


(5) ContentofC13ofeachorgan:Ci13(mg)=Ci×(Fi-Fnl)100×1000

F*_nl_*: no ^13^C labeling, natural abundance of ^13^C of each organ.


(6) C13partitioningrate:C13(%)=Ci13Cnetabsorption13×100%

### Dry Matter Weight and Root Morphology

After 30 d of treatment, samples were taken to measure the biomass of various organs of apple rootstocks. The rootstocks were divided into leaves, stems and roots. After being heated at 105°C for 30 min, it was dried at 80°C for 3 days. The dry matter weight of each organ was weighed with 1/1,000 electronic balance.

At the end of this experiment, three M9T337 rootstocks randomly sampled in each treatment group to analyze root morphology. The whole root system (root length, root surface area) was analyzed with WinRhizo software (WinRHIZO version 2012b, Regent Instruments Canada, Montreal, QC, Canada).

### Root Activity and Hormone Content in the Root

Root activities were measured using triphenyl tetrazolium chloride (TTC) method described by Chen et al. (2018). The root activity was determined by measuring the absorbance of ethanol at 485 nm.

The purified extraction product from 1.0 g of root (freeze-dried) was subjected to high-performance liquid chromatography (HPLC) analyses to determine the levels of indole-3-acetic acid (IAA), zeatin Riboside (ZR), gibberellic acid (GA_3_) and abscisic acid (ABA) as described by Almeida Trapp et al. (2014).

### Elemental Analysis

The dried leaves and roots were ground by an electric grinder, digested with H_2_SO_4_-H_2_O_2_, and the K content was determined by flame photometer. The contents of phosphorus, calcium (Ca) and magnesium (Mg) were determined by ICP-OES (ICP 6500 dual OES spectrometer, United States) after digestion with nitric acid–perchloric acid. The contents of N was determined by Kjeldahl apparatus (JK9870).

### Enzyme Activity

Rootstocks were harvested at 5-day intervals for 30 days for enzyme activities of carbon and nitrogen metabolism. NR activities were analyzed according to the method of Ding et al. (2006). The enzyme GS was measured by the method described by Hu et al. (2016). The enzyme NADH-glutamate synthase (NADH-GOGAT) was measured by the method described by Singh and Srivastava (1986) and the Fd-glutamate synthase (Fd-GOGAT) was measured by the method described by Migge et al. (1997).

The enzyme ribulose-1,5-bisphosphate carboxylase-oxygenase (Rubisco) was measured by the method described by Liu J. R. et al. (2013). The enzymes sucrose phosphate synthase (SPS) and sucrose synthase (SS) were extracted from frozen leaves as described previously (Huber and Israel, 1982). The enzyme phosphoenolpyruvate carboxylase (PEPC) was measured by the method described by Hu et al. (2017).

### Gas Exchange Parameters and Chlorophyll Fluorescence

Gas exchange parameters were measured at 5, 10, 15, 20, 25, 30 days after treatment. The *P*_n_, *G*_s_ and *C*_i_ were measured on the fourth main-stem leaf with a portable photosynthesis system (LI-6400, LI-COR Inc., United States) between 9:00 and 11:30 AM. Three rootstocks were selected for each treatment, and every measurement was repeated three times.

Chlorophyll fluorescence parameters were estimated on the same leaves using a pulse modulated chlorophyll fluorescence meter (PAM 2500, Walz, Germany) during the same period.

### RNA Extraction and Gene Expression by RT-qPCR

Nitrate (*NRT1.1, NRT1.2, NRT1.5, NRT2.4*) transporter and genes involved in N assimilation (*NR, GS1, NADH-GOGAT, Fd-GOGAT*) were selected for transcript analysis by RT-qPCR (reverse transcription and quantitative PCR). Total RNA was extracted using an RNAprep Pure Plant Kit (Tiangen, Beijing, China) according to the manufacturer’s instructions. The RNA was reverse-transcribed into cDNA using a RevertAid First Strand cDNA Synthesis Kit (TransGen) in a 20 μL reaction. The qPCR was performed in a 20 μL reaction mixture contained 10 μL of Green qPCR SuperMix, 1 μL of cDNA, 2 μL (1 μL of upstream and 1 μL of downstream primers) of primers and 7 μL of ddH_2_O. RT-qPCR assays were conducted using a CFX96 Real-Time PCR Detection system (BioRad, Hercules, CA, United States). The relative gene expression levels were calculated by the 2^–ΔΔCT^ method, and the *MdActin* gene was used as the internal control. These RT-qPCR experiments were performed with three technical replicates and three biological replicates. The primers used for RT-qPCR were listed in Table 1.

**TABLE 1:** Primer sequences for RT-qPCR.

Gene name	Forward sequence of the primers (5′→3′)	Reverse sequence of the primers (5′→3′)
*MdNRT1.1* (LOC103421872)	CTGGCTGGTCCCACAGTTCTT	CTTCATTCCTTTCGGGCACTC
*MdNRT1.2* (LOC103451876)	TTAATTGCTGCCACACTTCATAG	CACGATGTTTGGTTCTGATACTTC
*MdNRT1.5* (LOC103404033)	AACAAGACAATGCGACAG	GATGACAGTGACAACGATAC
*MdNRT2.4* (LOC103413242)	GCTGTACTCTTCCTGTGACTTT	CGTCGACTTCTCGACATCTTT
*MdNR* (LOC103439424)	GTCACACGAGTGGAGATAACAA	CAGAAACACCAGCACCAGTA
*MdGS1* (LOC103421902)	ATATCTGCTGGAGATGAACTGTGG	TGGACTTGGTGCTGTAGTTTGTG
*MdNADH-GOGAT* (LOC103443818)	ACTATGGTCGGTTCTCAAC	TCTTGATGCCTCTTGCTAA
*MdFd-GOGAT* (LOC114820893)	TTGAAGGAACTGGAGACC	GCAACATTTCTACCGACTT
*MdActin* (LOC103453508)	TGGTGTCATGGTTGGTATGG	CCGTGCTCAATGGGATACTT

### Data Analysis

The data presented as means (± SD). Statistical analyses of the data were performed using the SPSS (Statistics software, version 17.0, IBM, United States). Data were analyzed using one-way factorial analysis of variance (ANOVA) and a *post hoc* test (Duncan’s). Differences were considered significant at a probability level of *P* < 0.05. All data was drew using Origin 8.0 software.

## Results

### Effects of K Supply Level and Stability on Morphological Indices of M9T337 Rootstocks

**FIGURE 1:**
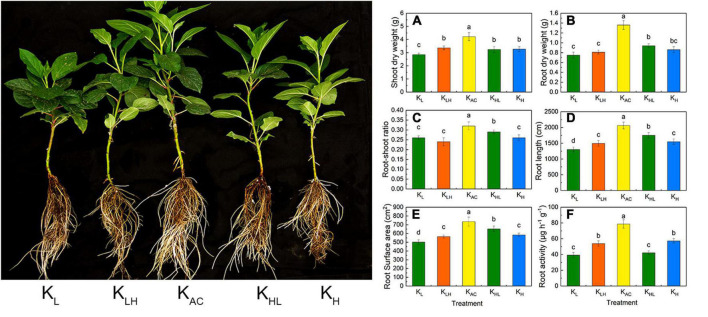
Shoot dry weight **(A)**, root dry weight **(B)**, root-shoot ratio **(C)**, root length **(D)**, root surface area **(E)** and root activity **(F)** of M9T337 rootstocks treated with different K treatment. Data show the means ± standard deviation of three independent samples. Different letters on vertical bars indicate significant differences (*P* < 0.05).

As shown in Figure 1, after 30 days of treatment, the shoot and root biomass of apple rootstocks was the highest in the K_AC_ treatment, and was the lowest in the K_L_ treatment. Deficit, excessive, and varied K application decreased the root biomass and root-to-shoot ratio. Further morphological analysis showed that K_AC_ treatment, followed by K_HL_ treatment, produced the best root development and the largest root length and root surface area. The K_L_ treatment produced the smallest root surface area. The determination of root activity showed that the K_L_ and K_HL_ treatments had the lowest root activity by the end of the experiment.

### Effects of K Supply Level and Stability on Endogenous Hormone Content in Roots of M9T337 Rootstocks

**FIGURE 2:**
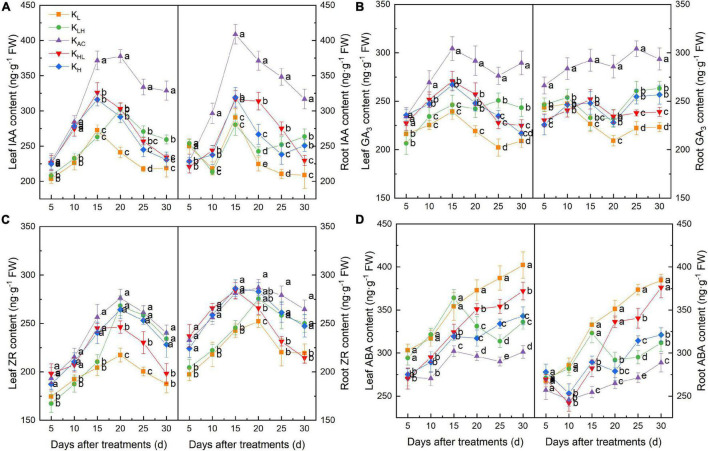
Effect of different K treatments on endogenous hormone content in roots and leaves of M9T337 rootstocks. Content of **(A)** IAA, **(B)** GA_3_, **(C)** ZR, and **(D)** ABA. Data show the means ± standard deviation of three independent samples. Different letters on vertical bars indicate significant differences (*P* < 0.05).

As shown in Figure 2, compared with other treatments, K_AC_ increased the IAA, GA_3_ and ZR contents, and decreased the ABA content in rootstock roots. Five days after treatment, the IAA content in the roots from K_L_ and K_LH_ treatments was significantly higher than in other treatments and then began to decrease, becoming significantly lower than that of the other treatments at 10 days. After 30 days, compared with the K_L_ treatment, the K_LH_ and K_H_ treatments had increased IAA and ZR contents and decreased GA_3_ and ABA contents in the roots. The hormone content in leaves was similar to that in roots, and the contents of IAA, GA_3_ and ZR in leaves were highest under K_AC_ treatment, whereas the content of ABA was the lowest.

### Effects of K Supply Level and Stability on the Element Content and Accumulation of M9T337 Rootstocks

**FIGURE 3:**
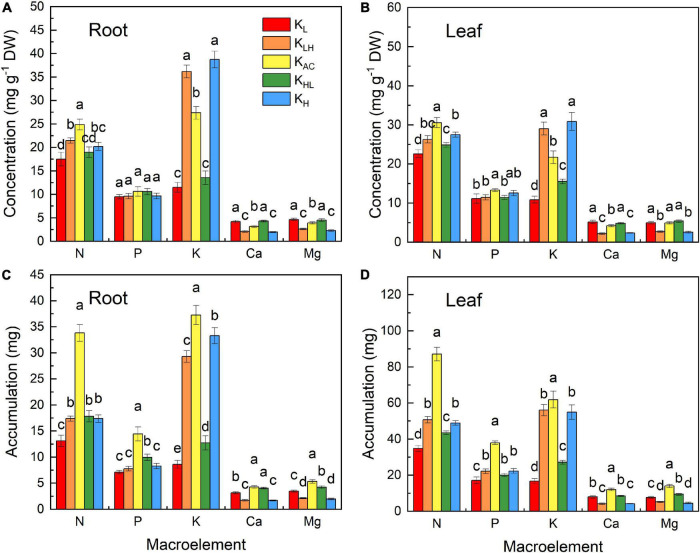
Effect of different K treatments on element contents in roots **(A)** and leaves **(B)**; element accumulation in roots **(C)** and leaves **(D)** of M9T337 rootstocks. Data show the means ± standard deviation of three independent samples. Different letters on vertical bars indicate significant differences (*P* < 0.05).

The content and accumulation of N, P, K, calcium (Ca) and magnesium (Mg) in plants were significantly affected by different K treatments (Figure 3). The K_AC_ treatment produced the highest N content and accumulation in the leaves and roots, while that in the K_L_ treatment was the lowest. K_H_ treatment significantly increased the K content but decreased the content and accumulation of Ca and Mg. In contrast, plants in the K_L_ treatment had increased Ca and Mg contents. The accumulation of N, P, K, Ca, and Mg was the highest in the K_AC_ treatment.

### Effects of K Supply Level and Stability on the Photosynthetic Characteristics of M9T337 Rootstocks

**FIGURE 4:**
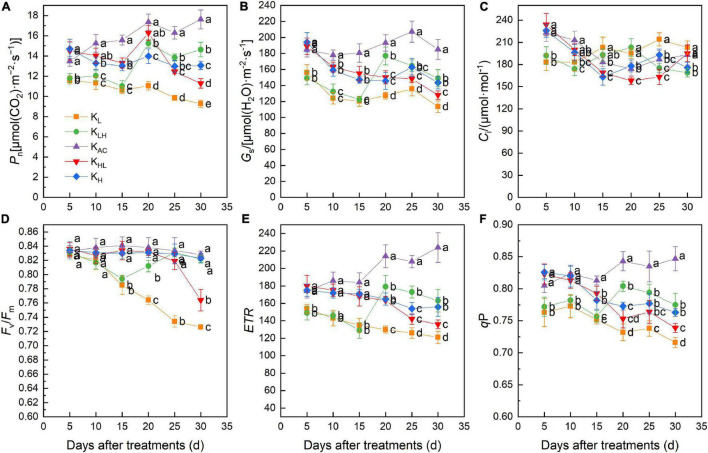
Effects of different K treatments on the *P*_n_ **(A)**, *G*_s_ **(B)**, *C*_i_ **(C)**, *F*_v_/*F*_m_ **(D)**, ETR **(E)**, *q*P **(F)** of M9T337 rootstocks. Data show the means ± standard deviation of three independent samples. Different letters on vertical bars indicate significant differences (*P* < 0.05). *P*_n_, net photosynthetic rate; *G*_s_, stomatal conductance; *C*_i_, intercellular CO_2_ concentration; *F*_v_/*F*_m_, maximum photochemical efficiency; ETR, electron transfer rate; *q*P, photochemical quenching coefficient.

Throughout the treatment period, the *P*_n_ and *G*_s_ of apple rootstock leaves were always at high levels under the K_AC_ treatment (Figures 4A,B). After 15 days of treatment, *P*_n_ was highest in the leaves under K_AC_, followed by K_H_ and K_HL_, and the *P*_n_ and *G*_s_ of leaves under K_L_ treatment were the lowest. However, the *C*_i_ values of K_L_ and K_HL_ treatments were significantly higher than those of the other treatments after 30 days (Figure 4C). These results indicated that K deficiency might damage the photosynthetic system of leaves.

Chlorophyll fluorescence parameters are often used to describe the photosynthetic physiological status of plants. After 15 days of treatment, the maximum photochemical efficiency (*F*_v_/*F*_m_), electron transfer rate (ETR) and photochemical quenching coefficient (*q*P) of PSII under K_L_, K_LH_, K_HL_ and K_H_ treatments were significantly lower than those of K_AC_ treatment. With the prolongation of treatment, *F*_v_/*F*_m_ of the leaves decreased significantly under K_L_ and K_HL_ treatments, while *F*_v_/*F*_m_ of the K_LH_ treatment returned to normal level after increasing K level (Figures 4D–F).

### Effects of K Supply Level and Stability on Rubisco, Sucrose Phosphate Synthase, Sucrose Synthase, and Phosphoenolpyruvate Carboxylase Activities of M9T337 Rootstocks

**FIGURE 5:**
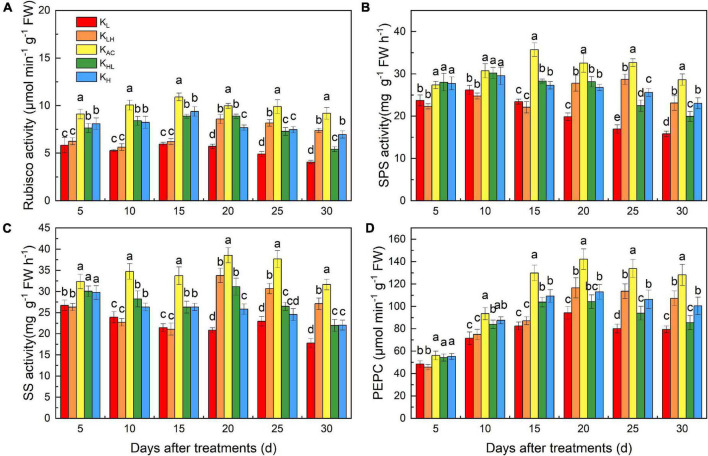
Effects of different K treatments on the Rubisco **(A)**, SPS **(B)**, SS **(C)** and PEPC **(D)** activities in the leaves of M9T337 rootstocks. Data show the means ± standard deviation of three independent samples. Different letters on vertical bars indicate significant differences (*P* < 0.05). Rubisco, ribulose-1,5-bisphosphate carboxylase-oxygenase; SPS, sucrose phosphate synthase; SS, sucrose synthase; PEPC, phosphoenolpyruvate carboxylase.

To further study the effects of different K treatments on C metabolism, we monitored C metabolism enzymes in the leaves of apple rootstocks at different stages (Figure 5). After 15 days of treatment, K_L_ and K_LH_ treatments had the lowest enzyme activities, and the K_AC_ treatment had the highest enzyme activity. Rubisco, SPS, SS and PEPC activities in the K_LH_ treatment were significantly higher than those in the K_L_ treatment after 20 days (5 days after K supply change), whereas those in the K_HL_ treatment were significantly lower than those of K_H_ treatment. Throughout the treatment period, the activities of Rubisco, SPS, SS and PEPC were highest under the K_AC_ treatment.

### Effects of K Supply Level and Stability on Accumulation and Distribution of ^13^C and ^15^N in Different Organs of M9T337 Rootstocks

**FIGURE 6:**
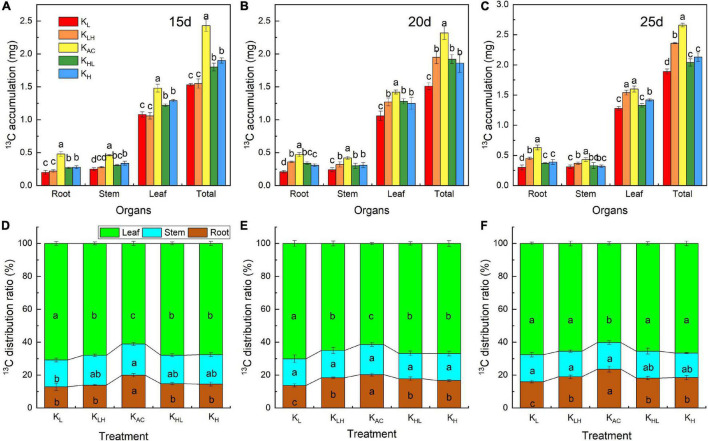
Effects of different K treatments on ^13^C accumulation **(A)** and ^13^C distribution ratio **(D)** after 15 days treatments, ^13^C accumulation **(B)** and ^13^C distribution ratio **(E)** after 20 days treatments and ^13^C accumulation **(C)** and ^13^C distribution ratio **(F)** after 25 days treatments of M9T337 rootstocks. Data show the means ± standard deviation of three independent samples (three rootstocks as a sample). Different letters on vertical bars indicate significant differences (*P* < 0.05).

**FIGURE 7:**
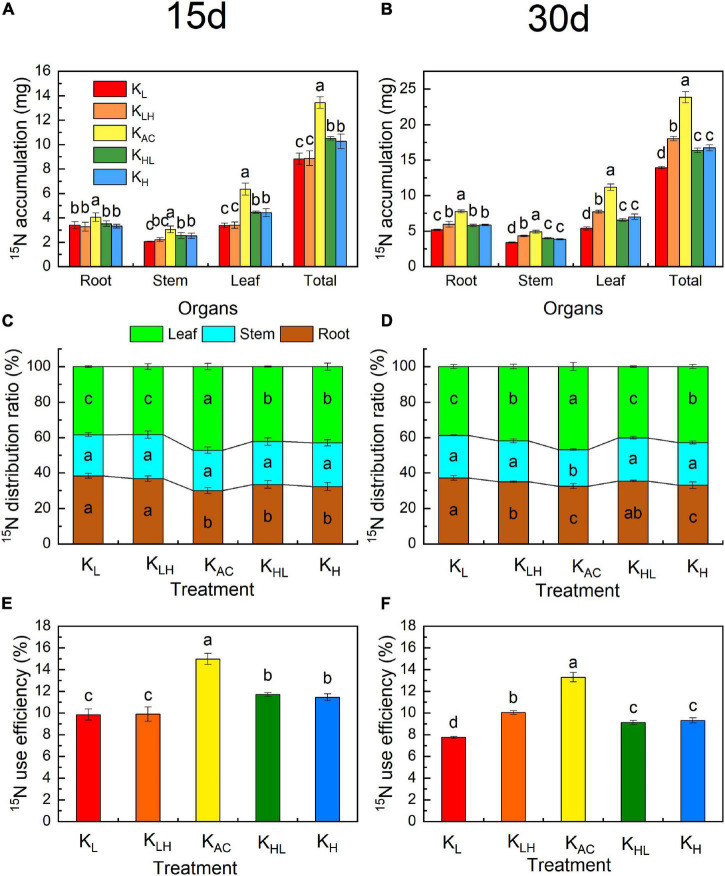
Effects of different K treatments on ^15^N accumulation **(A)**, ^15^N distribution ratio **(B)** and ^15^NUE **(C)** after 15 days treatment and ^15^N accumulation **(D)**, ^15^N distribution ratio **(E)** and ^15^NUE **(F)** after 30 days of M9T337 rootstocks. Data show the means ± standard deviation of three independent samples (three rootstocks as a sample). Different letters on vertical bars indicate significant differences (*P* < 0.05).

We used the ^13^C and ^15^N stable isotope technique to further analyze the absorption and distribution of C and N in apple rootstocks under different K supply levels and stability treatments (Figures 6, 7). The highest accumulation of ^13^C in each organ of the rootstocks was under K_AC_ treatment at different marker periods, indicating that the C assimilation and accumulation were the strongest under an appropriate and constant K supply. After 15 days of treatment, the ^13^C allocation rates under K_L_, K_LH_, K_AC_, K_HL_ and K_H_ treatments were 12.87, 13.94, 19.90, 14.79, and 14.54%, respectively (Figure 6D). The ^13^C allocation rate in the K_AC_ treatment was the highest, and that of K_L_ was the lowest. After 25 days of treatment, however, the ^13^C allocation rate of roots arranged from high to low in order was K_AC_, K_LH_, K_H_, K_HL_, and K_L_ (Figure 6E). Compared with the K_L_ treatment, the root ^13^C allocation rate in the K_LH_ treatment increased significantly. Contrary to the rule of root ^13^C allocation rate, the ^13^C allocation rate of leaves under K_AC_ treatment was the lowest, whereas that in the K_L_ treatment was the highest.

After 15 days of treatment, K_AC_ resulted in the largest accumulation of ^15^N (Figure 7A), the highest utilization rate of ^15^N (Figure 7C), and the highest ^15^N allocation rate in leaves (Figure 7B). The ^15^N allocation rate of rootstock leaves was the lowest, whereas that of roots was the highest under low K treatment. After 30 days of treatment, the accumulation of ^15^N under different treatments from high to low in order was K_AC_, K_H_, K_LH_, K_HL_, and K_L_. The utilization rate of ^15^N was still the highest under the K_AC_ treatment. Compared with the K_L_ treatment, K_LH_ treatment increased ^15^N distribution in leaves and the ^15^N utilization rate, whereas K_HL_ treatment decreased ^15^N distribution in leaves and the ^15^N utilization rate compared with the K_H_ treatment.

### Effects of K Supply Level and Stability on Nitrate Reductase, Glutamine Synthetase, NADH-GOGAT and Fd- GOGAT Activities and Gene Expression of M9T337 Rootstocks

**FIGURE 8:**
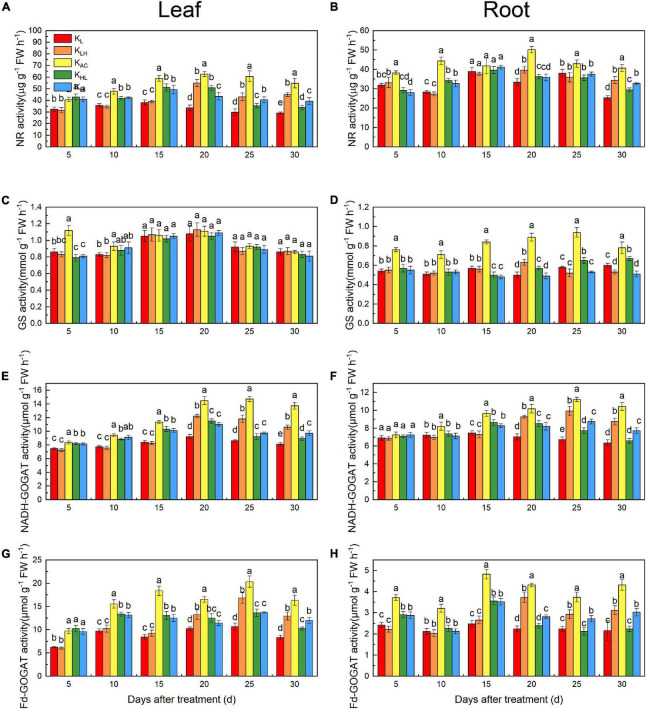
Enzyme specific activities in leaves and roots: NR **(A,B)**, GS **(C,D)**, NADH-GOGAT **(E,F)**, Fd-GOGAT **(G,H)** of M9T337 rootstocks treated with different K treatments. Data show the means ± standard deviation of three independent samples. Different letters on vertical bars indicate significant differences (*P* < 0.05).

As shown in Figure 8, the NR activity in roots and leaves was significantly reduced by K_L_, K_H_, K_LH_ and K_HL_ conditions, and GS activity in roots decreased more significantly under high K conditions (K_LH_, K_H_). After 15 days, the NADH-GOGAT and Fd-GOGAT activities in the K_LH_ treatment was higher than that of the K_HL_ treatment, and it was higher under K_H_ treatment than under K_L_ treatment, indicating that a low K supply more significantly inhibited GOGAT activity.

**FIGURE 9:**
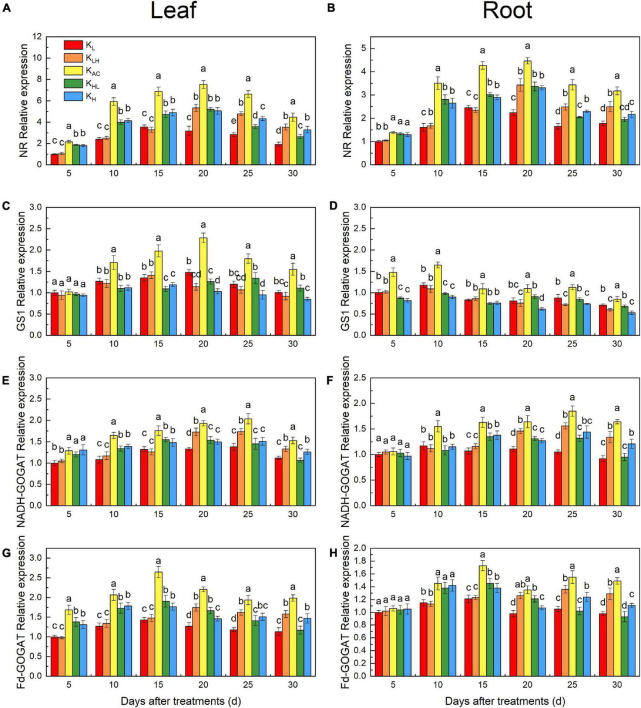
NR relative expression in the leaves **(A)** and roots **(B)**; GS1 relative expression in the leaves **(C)** and roots **(D)**; NADH-GOGAT relative expression in the leaves **(E)** and roots **(F)**; Fd-GOGAT relative expression in the leaves **(G)** and roots **(H)** of M9T337 rootstocks treated with different K treatments. Data show the means ± standard deviation of three independent samples. Different letters on vertical bars indicate significant differences (*P* < 0.05).

We also measured the expression of *MdNR*, *MdGS1*, *MdNADH-GOGAT* and *MdFd-GOGAT* in roots and leaves. The *MdNR* expression in high K treatment was higher than that in low K treatment, and lower than that in the K_AC_ treatment (Figures 9A,B). This is the same as the results of enzyme activity. The expression of *MdNADH-GOGAT* and *MdFd-GOGAT* was similar to that of *MdNR* (Figures 9E–H). However, the expression of *MdGS1* in leaves and roots was significantly inhibited by high K treatment (Figures 9C,D). The expression of *MdNR*, *MdGS1*, *MdNADH-GOGAT* and *MdFd-GOGAT* in K_AC_ treatment was significantly higher than that in other treatments.

### Effects of K Supply Level and Stability on MdNRT1.1, MdNRT1.2, MdNRT1.5 and MdNRT2.4 Expression in M9T337 Rootstocks

**FIGURE 10:**
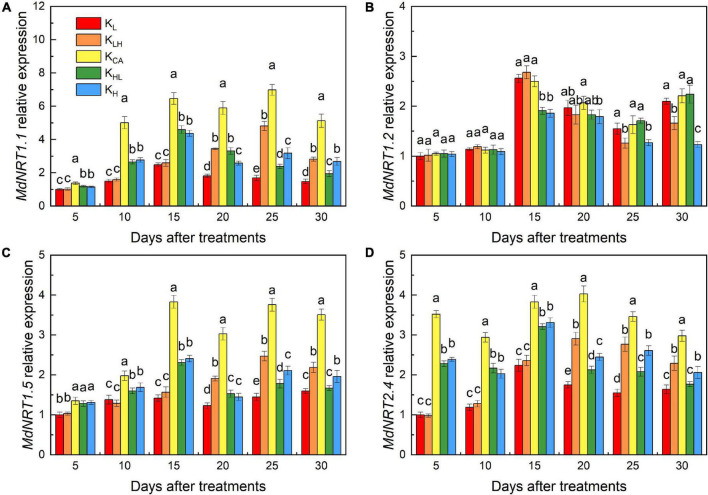
Effects of different K treatments on *MdNRT1.1* gene expressions **(A)**, *MdNRT1.2* gene expressions **(B)**, *MdNRT1.5* gene expressions **(C)** and *MdNRT2.4* gene expressions **(D)** in the roots of M9T337 rootstocks. Data show the means ± standard deviation of three independent samples. Different letters on vertical bars indicate significant differences (*P* < 0.05).

There was significant difference in the expression of *MdNRT1.1, MdNRT1.5 and MdNRT2.4* after 5 days of treatment (Figures 10A,C,D). Five days after treatment, the expression of *MdNRT1.1* and *MdNRT2.4* in the roots under the K_AC_ treatment began to be significantly higher than other treatments, and lasted until the end of the experiment. After 30 days of treatment, the expression of *MdNRT1.1, MdNRT1.5 and MdNRT2.4* in roots of high K treatment (K_LH_, K_H_) was significantly higher than that of low K treatment (K_L_, K_HL_), but the relative expression of *MdNRT1.2* in high K treatment was significantly lower than that in low K treatment (Figure 10B).

## Discussion

### Changes in Growth, Endogenous Hormones and Nutrient Element Content of M9T337 Rootstocks Under Different K Treatments

The level and mode of K supply affected the growth of apple rootstocks. The root and shoot biomass under the K_AC_ treatment was significantly higher than that of the other treatments. Root growth is controlled by several factors, the most important of which are hormones and nutrition (Osmont et al., 2007; Qi et al., 2019). In our study, both unsuitable and unstable K supply significantly inhibited the root growth of rootstocks, and this was related to the lower IAA, GA_3_, and ZR contents and higher ABA content in roots. Zhao et al. (2016) observed similar results in maize. The changes in hormone content in the leaves were similar to those in the roots. In the K_AC_ treatment, higher IAA, GA_3_, and ZR contents in leaves also promoted shoot growth.

The normal growth of plants is inseparable from the coordinated supply of mineral elements. We found that the accumulation of N, P, K, Ca, and Mg in M9T337 rootstocks under K_AC_ treatment was significantly higher than that in other treatments after 30 days of culture. The results indicated that K_AC_ promoted the absorption and utilization of nutrients by plants, which may have been related to the stronger root activity and larger root surface area. K_L_ treatment and K_HL_ treatments reduced the content of N and K and increased the content of Ca and Mg in rootstocks. However, the content and accumulation of Ca and Mg in rootstocks were decreased under K_L_ and K_HL_ treatments. The results showed that a high K addition could inhibit the absorption of Ca^2+^ and Mg^2+^, similar to the results of previous studies (Farhat et al., 2013; Okturen Asri and Sonmez, 2016). Chlorophyll, photosynthesis, movement of the stoma, and transpiration are all affected and regulated by Ca and Mg (Tan et al., 2011; Tränkner et al., 2018), so the decrease in growth under high K treatment may be related to decreases in Ca and Mg contents in rootstocks.

### Differences in Photosynthetic Fluorescence Characteristics and C Metabolism of M9T337 Rootstocks Under Different K Treatments

As the main osmoregulation substance of guard cells, K plays an important role in photosynthesis (Xie et al., 2020), and its abundance affects stomatal function (Hou et al., 2018). The *G*_s_ under the low K treatment was lower than that of the high K treatment, which may be related to the higher ABA content in leaves under low K conditions (Webb and Hetherington, 1997). Five days after the K supply level change, ABA content in the leaves of the K_LH_ treatment decreased sharply and *G*_s_ increased significantly, supporting this hypothesis. According to Farquhar and Sharkey (1982), the results of gas exchange parameters indicated that the decrease of *P*_n_ under low K treatment might be caused by non-stomatal factors; thus, we also measured the chlorophyll fluorescence of rootstock leaves. The level of *F*_v_/*F*_m_ is an important indicator to measure whether the photosynthetic system of leaves is damaged (Singh et al., 2013). K_L_ treatment significantly reduced *F*_v_/*F*_m_, which is consistent with the results of Lu et al. (2015) on rape. However, *F*_v_/*F*_m_ returned to normal levels when the K supply level changed from low to high, indicating that the inhibition of photosynthesis by the low K treatment was more serious than that by the high K treatment. Compared with the other treatments, the *F*_v_/*F*_m_, *ETR* and *qP* of leaves under K_AC_ treatment were the highest, indicating that an appropriate and constant K supply can improve the photosynthetic efficiency of apple rootstocks, optimize the processes of photosynthetic phosphorylation and electron transfer, and keep the reaction center of leaves open. These effects are conducive to the improvement of photosynthesis and carbon assimilation efficiency.

K plays an important role in the assimilation and transport of photosynthetic products (Zahoor et al., 2017; Tränkner et al., 2018). The ^13^C isotope labeling results showed that the K_AC_ treatment significantly promoted the C assimilation and accumulation and the transportation of photosynthate from leaves to roots. This is beneficial to the growth of roots. Rubisco, SPS, SS and PEPC are key enzymes in the assimilation and transportation of photosynthate (Lan et al., 2020). Five days after the K supply level changed, Rubisco, SPS, and SS activities in the leaves of seedlings under unstable K treatment were significantly higher than those under K_L_ and K_H_ treatments, but they were still significantly lower than those under the K_AC_ treatment. Rubisco, SPS, and PEPC activities in leaves of seedlings treated with K_HL_ were significantly lower than those under K_H_ treatment at 10 days after the K supply level changed. This shows that K_HL_ treatment can only alleviate the negative effects of high K conditions after a short exposure time. Throughout the treatment period, Rubisco, SPS, SS and PEPC activities were significantly higher in the K_AC_ treatment than in the other treatments. Therefore, the ^13^C accumulation and ^13^C distribution rate in roots under an unstable K supply were lower than those under an appropriate and constant K supply. An appropriate and constant K supply was best for both photosynthesis and photosynthate transport.

### Differences in N Metabolism of M9T337 Rootstocks Under Different K Treatments

K also strongly affects the absorption, assimilation and distribution of N (Coskun et al., 2017b). In our study, the N content and ^15^N accumulation and utilization efficiency of apple rootstocks were significantly reduced under low, high and unstable K supply conditions. This may have been related to the different activities of enzymes and gene expression related to N metabolism under different K treatments. We found that the activities and transcriptional levels of NR, GS and GOGAT were significantly higher under K_AC_ treatment, thus promoting the assimilation and utilization of N by rootstocks. Hou et al. (2019) obtained similar results in rice. Compared with K_L_ and K_H_ treatments, an unstable K supply improved the activities of related N-metabolizing enzymes and their transcriptional levels in a short period of time, but there was still a large difference compared with the K_AC_ treatment. Another reason for the increase of NO_3_^—^ uptake under K_AC_ treatment may be related to the higher expression of NRTs. *MdNRT1.1*, *MdNRT1.2* and *MdNRT2.4* are important NRTs and are mainly involved in NO_3_^–^ uptake in the roots (Xu et al., 2012). The K_AC_ treatment significantly increased *MdNRT1.1, MdNRT1.2* and *MdNRT2.4* expression in the roots of apple rootstocks, which was conducive to enhancing NO_3_^–^ uptake. Low K reduced the transcription levels of *MdNRT1.1* and *MdNRT2.4* in rootstock roots, similar to the results obtained in *Arabidopsis* (Armengaud et al., 2009). High K treatment inhibited the absorption of Ca, resulting in Ca deficiency. Ca deficiency can reduce the activities of N metabolism enzymes and NRT gene expression (Xing et al., 2021). Therefore, high K treatment may have had adverse effects on N absorption through Ca antagonism.

In addition, the N distribution in the plant also affected the N absorption efficiency. The results of ^15^N tracing showed that the ^15^N allocation rate of rootstock leaves was highest under the K_AC_ treatment, which may be related to the increase of *MdNRT1.5* transcription. *MdNRT1.5* participates in NO_3_^–^ and K^+^ loading of the xylem and plays an important role in regulating NO_3_^–^ and K^+^ transport from the roots to shoots (Chen et al., 2021). Increasing the distribution of NO_3_^–^ in the upper part of the plant can make full use of solar energy for NO_3_^–^ metabolism and energy conversion, thereby improving NUE (Han et al., 2016). Therefore, the increase in NUE under an appropriate and constant K supply may be related to the increase in N allocation in the shoots.

## Conclusion

**FIGURE 11:**
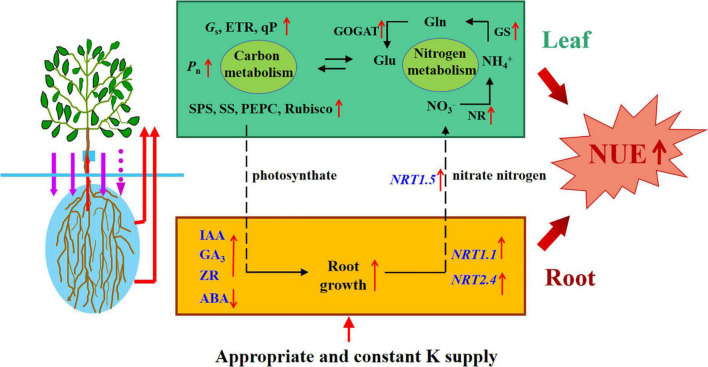
Schematic model displaying the role of K on apple rootstock growth and Nitrogen and carbon metabolism. Appropriate and constant K supply promoted root growth and increased the activities of carbon and nitrogen metabolism enzymes, which promoted the N absorption and assimilation, meanwhile, thus increased the NUE of apple rootstocks.

Compared with an unsuitable and unstable K supply, an appropriate and constant K supply could (i) maintain the balance of endogenous hormones and nutrient elements in plants; (ii) enhance the enzyme activities of C and N metabolism; (iii) upregulate the transcript levels of genes involved in N uptake and assimilation; and (iv) optimize ^13^C and ^15^N allocation within rootstocks. In summary, our results demonstrate that the K supply method led to significant differences in endogenous hormones and C and N nutrition; an appropriate and constant K supply can promote the growth of apple rootstocks by optimizing hormone levels and C and N metabolism (Figure 11). This study provided a scientific basis for fertilization and improving N fertilizer utilization rates in apple production.

## Data Availability Statement

The original contributions presented in the study are included in the article/supplementary material, further inquiries can be directed to the corresponding author/s.

## Author Contributions

YJ, SG, and XX conceived and designed the experiments. XX, FW, YX, JL, ML, XD, and ZZ performed all experiments. XX, ZZ, and SG analyzed the data and wrote the manuscript. All authors contributed to the article and approved the submitted version.

## Conflict of Interest

The authors declare that the research was conducted in the absence of any commercial or financial relationships that could be construed as a potential conflict of interest.

## Publisher’s Note

All claims expressed in this article are solely those of the authors and do not necessarily represent those of their affiliated organizations, or those of the publisher, the editors and the reviewers. Any product that may be evaluated in this article, or claim that may be made by its manufacturer, is not guaranteed or endorsed by the publisher.
